# Mandibular prognathism attenuates brain blood flow induced by chewing

**DOI:** 10.1038/s41598-019-55553-5

**Published:** 2019-12-13

**Authors:** Hiroyuki Kanzaki, Satoshi Wada, Masao Kumazawa, Yuko Yamada, Tomomi Sudo, Erika Ozawa, Takuya Seko, Shun Akaike, Masumi Murakami, Takashi Oikawa, Satoshi Okumura, Yoshiki Nakamura, Hiroshi Tomonari

**Affiliations:** 1 Department of Orthodontics, Tsurumi University, School of Dental Medicine, Yokohama, Japan; 2 Department of Physiology, Tsurumi University, School of Dental Medicine, Yokohama, Japan

**Keywords:** Near-infrared spectroscopy, Dementia, Malocclusion, Craniofacial orthodontics, Brain imaging

## Abstract

Mastication is closely related to brain function. Animal experiments have revealed that tooth loss has a negative influence on brain function. Clinical studies also suggest that normal occlusion is an essential factor for favorable brain function. Mandibular prognathism (MP) usually results in occlusal dysfunction. However, the relationship between MP and brain function remains unclear. In the present study, we examined the relationship between MP and brain function by measuring brain blood flow (BBF). Seventeen subjects with normal occlusion (NORM) and 25 patients with MP participated in this study. The number of occlusal contacts were counted. Electromyography of the masseter muscles during clenching was also recorded. BBF was measured with non-invasive functional near-infrared spectroscopy during calculation task and chewing task. The number of the occlusal contacts and masseter muscle activity were lower in MP compared with NORM. The calculation task increased BBF in both groups. The chewing task also increased BBF in the inferior frontal gyrus in both groups, although the increase in MP was smaller than in NORM. We discovered that patients with MP exhibited a smaller increase in BBF at the inferior frontal gyrus during chewing as compared with NORM. As such, MP would negatively affect brain function.

## Introduction

Mastication is an essential function^1^ and is a coordinated effort between the occlusal unit, masticatory muscle, sensory system, and brain^2^. Sensorimotor neurons synapse with trigeminal motoneurons involved in specific glossomandibular movements^3^. Conversely, ascending signals from the periodontal mechanoreceptor via the trigeminal nerve induce an extensive arousing signal in the brain^4^. Therefore, mastication is closely related to brain function.

Tooth loss has been shown to reduce *trkB* mRNA expression in the rat brain, which accelerated spatial memory impairment^5^. In addition, reduced masticatory sensory input due to long-term soft-diet feeding induced neuron loss and reduced memory/learning ability in mice^6^. Occlusal stimuli are critical to maintaining brain function in experimental animals^7^, and artificial occlusal disharmony impairs spatial memory and induces neuron degeneration in mice^8^.

Human clinical studies also support the connection between mastication and brain function. Prosthodontic improvement of occlusion markedly recovered the frontal lobe function during mastication^9^. Furthermore, denture wearing contributed to an improvement to the clinical dementia rating^10^. A study investigating the interaction between mastication and brain blood flow (BBF) with positron-emission tomography revealed that chewing activates widespread regions of the brain^11^. Electroencephalograms also revealed that experimental premature contact affects brain function in healthy volunteers^12^. Together, ideal occlusion would be an essential factor for maintaining favorable brain function.

Patients with jaw deformities, such as mandibular prognathism (MP), show reduced occlusal function through^13^ extensive failure in the occlusal contacts^14^ and a reduction of bite force^15^. As such, jaw deformity is presumed to have a negative influence on brain function due to decreased occlusal function. However, to date, there has been no investigation on the relationship between MP and brain function. In the present study, we examined BBF to investigate the relationship between MP and brain function using functional near-infrared spectroscopy (fNIRS).

## Results

### The number of occlusal contacts in MP was smaller than in NORM

First, we examined the negative impact of MP on occlusal function. The number of the occlusal contacts in MP was smaller than that observed in NORM (Table 1), signifying MP has a negative influence on occlusal function.

**Table 1:** The number of occlusal contact in each groups.

Groups	Mean	SE	Significance versus NORM
NORM (N = 17)	25.4	1.8	—
MP (N = 25)	16.1	1.2	P < 0.01

Statistical difference was tested using Student’s t-test.

### Masseter and temporal muscle activity during the clenching task was weaker in MP than reported with normal occlusion

We then compared masseter and temporal muscle activity during the clenching task using Electromyography (EMG), to clarify the functional negative impact of MP. The mean EMG value for masseter muscle activity during clenching was 159.8 ± 34.8 μV in MP (Table 2), smaller than that previously reported in normal occlusion (266.1 ± 30.0 μV)^16^. Similarly, the EMG value for the anterior part of temporal muscle activity during clenching in MP (232.8 ± 12.8 μV) was smaller than that in normal occlusion (324.4 ± 28.0 μV).

**Table 2:** EMG value of masseter and temporal muscle during clenching in each groups.

Groups	Mean	SE	Significance versus
**Masseter muscle**			
Normal occlusion (N = 11)	266.1	30.0	—
MP (N = 25)	159.8	34.8	P < 0.05
**Anterior part of temporal muscle**			
Normal occlusion (N = 11)	324.4	28.0	—
MP (N = 25)	232.8	12.8	P < 0.05

The data of Normal occlusion group were from the reference No. 16.

Statistical difference was tested using Student’s t-test.

These data suggest MP has a negative influence on occlusal function, particularly from the point of occlusal force. Combining the results of the occlusal contact and masseter and temporal muscle activity, the patients with MP seem to be in the failure of the occlusal function.

### The calculation task increased oxy-Hb in both groups

**Figure 1:**
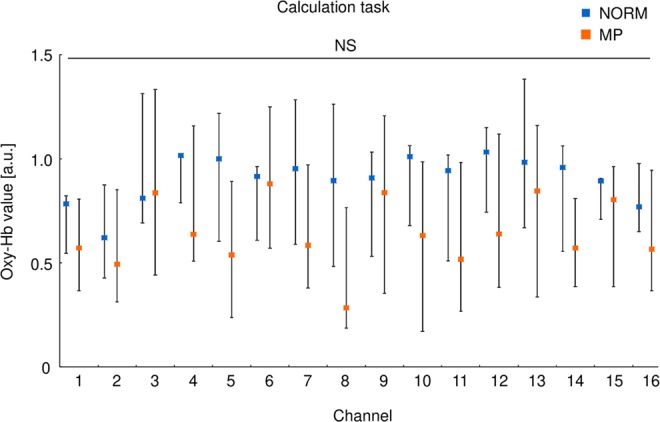
Oxy-Hb values during the calculation task. The value at each channel expressed as median and quartiles in each group. NORM: blue square, MP: orange square. NS: no significant difference between the groups (Mann-Whitney U test).

The amount of oxy-Hb during the calculation task was measured in all subjects (Fig. 1). We observed increased oxy-Hb in almost all channels, including the anterior prefrontal cortex^17^. There was no difference in the BBF, inferred from oxy-Hb levels, between groups during the calculation task. We observed no difference in calculating ability between the groups (data not shown). These results suggest that the calculation task induces a certain amount of BBF, regardless of differences in occlusion.

### Chewing tasks increased BBF to a greater extent in NORM than in MP

**Figure 2:**
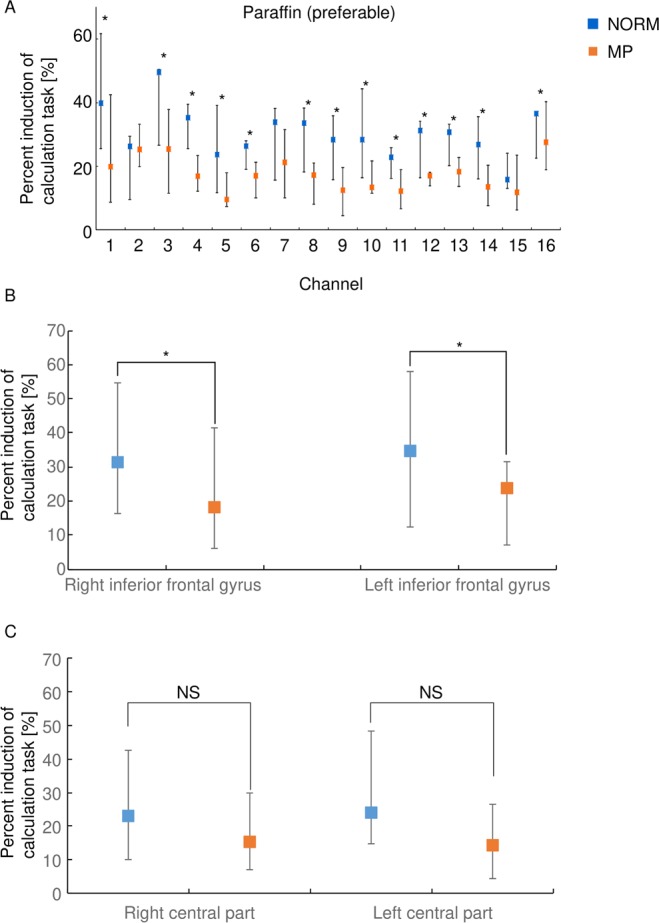
The percent induction of oxy-Hb value against the calculation task. (**A**) Percent induction of Oxy-Hb values against calculation task during chewing of the paraffin on the preferred side are shown. The values are expressed as median and quartiles in each group. *P < 0.05 between the groups (Mann-Whitney U test). (**B**) Percent induction of Oxy-Hb values at the right and the left inferior frontal gyrus during chewing of the paraffin on the preferred side are shown. The values are expressed as median and quartiles of the channels 1 to 4 (right) and 13 to 16 (left) in each group. *P < 0.05 between the groups (Mann-Whitney U test).

Next, we examined the influence of chewing on BBF compared to the calculation task (Fig. 2A). Chewing increased BBF in both groups, but to a lesser extent to the increases observed during the calculation task. Next, we determined whether there were any differences in chewing-induced BBF between NORM and MP groups. The induction of BBF by chewing was higher in NORM than in MP, with significant differences between groups in some channels (Chs 1, 3, 4, 5, 6, 8, 9, 10, 11, 12, 13, 14, and 16).

We then focused on the inferior frontal gyrus, which was reported to play an important role in cognitive function^18,19^. The chewing-induced increases in BBF at the inferior frontal gyrus were high in NORM compared with MP (Fig. 2B). On the other hand, the chewing-induced increase in BBF at the other part of brain, such as right and left central parts, exhibited no statistical difference between the groups (Fig. 2C).

These results suggest that the chewing task increased BBF at the inferior frontal gyrus, and that this induction was higher in NORM compared with MP.

### Case report

**Figure 3:**
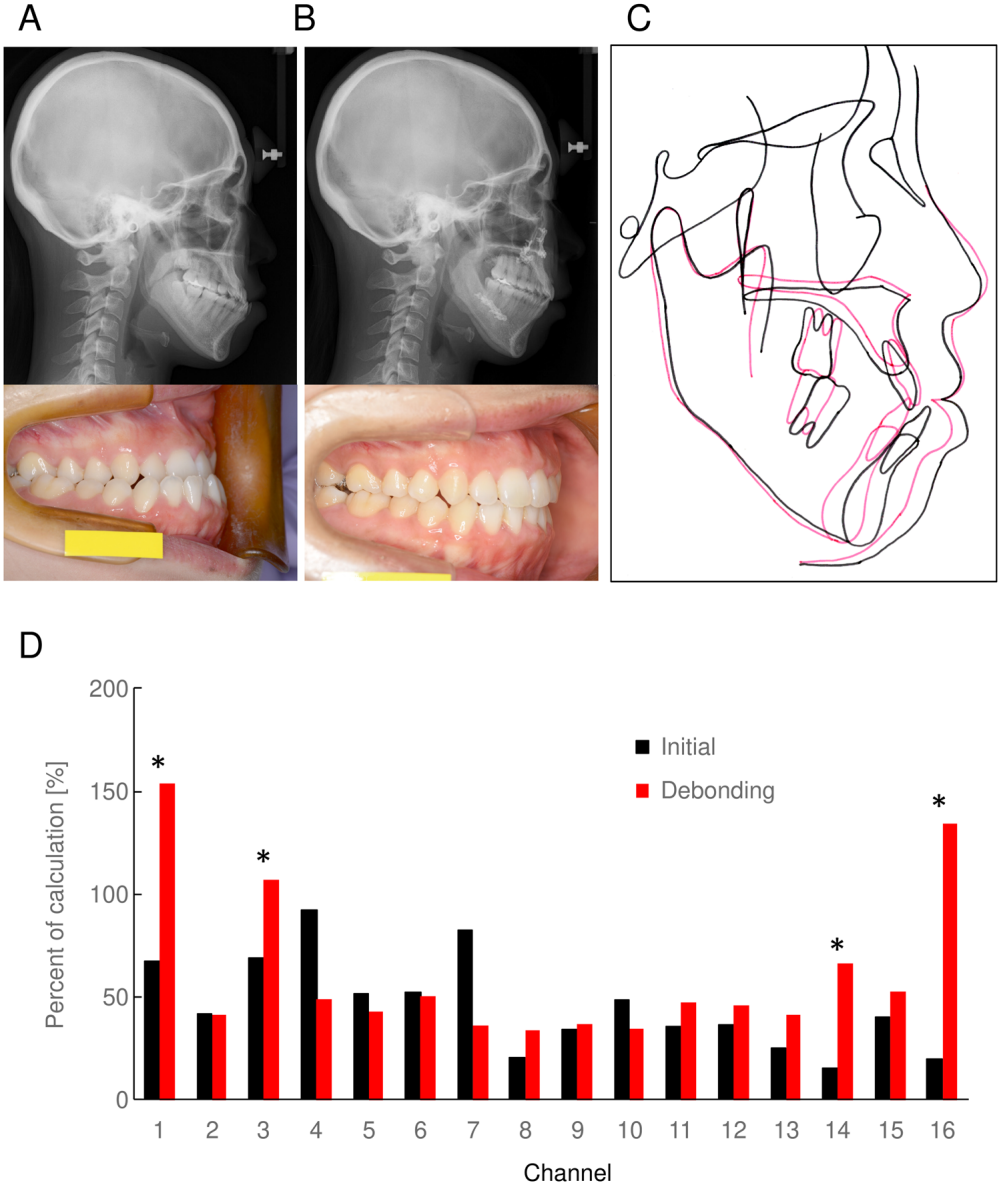
Case presentation of surgical orthodontic treatment. Cephalometric radiograph and intraoral photograph at initial examination (**A**) and at debonding (**B**) are shown. (**C**) Superimposition of tracings of cephalometric radiograph at initial examination (black) and at debonding (red) is shown. (**D**) BBF induced by chewing at initial (black) and debonding (red) in each channels are shown. Percent of calculation task are shown. *P < 0.05 between the groups.

The Japanese female patient aged 33-years-old presented mandibular prognathism (Fig. 3A). Cephalometric analysis indicated a severe skeletal Class III malocclusion (increased SNB angle of 83.1). She had minor crowding in upper and lower dentition. The patient was diagnosed with an Angle Class III malocclusion with severe skeletal Class III. The treatment objectives were as follows: (1) to correct minor crowing in upper and lower dentition without premolar extraction, (2) to correct skeletal Class III and improve the prognathic appearance of the facial profile by two jaws surgery (impaction of posterior part of maxilla by Le Fort I osteotomy, and mandibular setback by sagittal split ramus osteotomy (SSRO)), (3) to establish good functional occlusion by achieving an Angle Class I occlusion, and ideal overjet and overbite.

### Treatment outcomes

Cephalometric radiograph and intraoral photographs at debonding indicate the improved antero-posterior skeletal problem and establishment of good functional occlusion (Fig. 3B). Crossbite and crowding were corrected, and good intercuspation with an Angle Class I molar relationship was observed. Superimposition of cephalometric tracings indicates that significant improvement of the profile (Fig. 3C). No of occlusal contact at initial was 7, and it was increased to 14 at the end of surgical orthodontic treatment. Of interest, BBF was increased by surgical orthodontic treatment at some channels in inferior frontal gyrus (Fig. 3D).

## Discussion

fNIRS clearly demonstrated the changes in BBF that occurred during the study tasks. BBF during the calculation task was always higher than BBF during the chewing task. This is consistent with the report of calculation tasks inducing more cortical activation than control tasks due to coordinated higher level brain function^20^. There was no difference in BBF between groups during the calculation task. Therefore, in this study we used the calculation task as the positive control and calculated the ratio of BBF during the chewing task compared with the calculation task. Furthermore, this normalization by the calculation task would compensate the possible change of BBF by time variation. Considering that the levels of BBF during the calculation task were similar between groups, there is no influence of MP in regards to the calculation task. Indeed, we observed no difference in calculating ability between the groups.

In this study, we discovered that chewing tasks increased BBF in both groups. BBF increased at the inferior frontal gyrus, consistent with previous reports that induction of BBF by chewing was observed in the prefrontal and posterior parietal cortex areas using fMRI^21^ and fNIRS^22^. This consistency confirms fNIRS is a useful tool to examine BBF changes in the prefrontal cortex area induced by chewing. As to the reproducibility of response of brain blood flow by chewing, we firstly confirmed a certain level of reproducibility even after a week (The values of ICC were over 0.7). In addition, we set the several tasks of chewing other than preferential paraffin wax chewing, such as right and left side chewing of paraffin wax, and preferential hard gummy chewing. Each chewing tasks basically increased brain blood flow similar to the preferential paraffin wax chewing, though the extent of the increase was different among the tasks (Data not shown). The values of ICC in our research were over 0.7, and this value was similar to that of Plichta’s paper (ICCs: up to 0.84)^23^. We presumed that our methodology to measure the change of brain blood flow by NIRS is accurate enough for our study.

The increases in BBF by chewing observed in the inferior frontal gyrus were higher in NORM compared with MP, which indicates possible retardation in normal inferior frontal gyrus function. As to the median of the absolute value of oxy Hb in Chs-1 to 4 were 0.4375, 0.1821, 0.3166, and 0.2687 in NORM, and 0.1559, 0.1570, 0.2061, and 0.1718 in MP, respectively. Therefore, we presumed that though large variance between the channels widen the quartiles of the right and the left inferior frontal gyrus and furthermore, reduce the difference between the groups, the increase in BBF induced by chewing was retarded in MP as compared to that in NORM. The inferior frontal gyrus has been reported to play an important role on cognitive function^18,19^. As such, our study indicates MP may negatively influence this cognitive function. Several literatures indirectly support this hypothesis. The survey of the 41 persons retaining 20 or more teeth after the age of 80 in Chiba, Japan, exhibit no MP, and it concluded that the normal skeletal pattern would be mandatory to maintain favorable occlusion at advanced age^24^. The linkage between cognitive impairment and the loss of the teeth in elderly Japanese population was reported^25^. Together, MP would complicate to achieve the situation of retaining 20 or more teeth after the age of 80, which result in the onset of cognitive impairment.

NIRS is used as an ancillary equipment for diagnosis of psychological problem such as depression, and negative correlation between the depression severity and frontal and right temporal activations^26^. The extent of reduction of brain blood flow by depression as compared to that in the healthy subject was about the half^27^. Depression is considered to the risk factor for cognitive impairment such as Alzheimer disease^28^, and linkage between cerebral blood flow dysregulation and the risk for cognitive impairment was also reported^29^. Comparing this extent of reduction in brain blood flow by depression, MP-mediated reduction of the chewing induced brain blood flow was weak. Therefore, we speculate that MP would give certain amount of negative impact to brain function though the extent is relatively less as compared to depression.

Prefrontal cortex activity is closely related to occlusal function^9^. The difference in BBF between NORM and MP is dependent upon chewing ability, i.e., the difference in occlusal function, since the number of contact points in MP is significantly less than that in NORM^30^. We also found a smaller number of occlusal contacts in MP compared with NORM. The change of BBF by chewing seemed to correlate with the number of occlusal contacts. It was reported that BBF was closely related to sensory function in the periodontal ligament (PDL)^31–33^. Narita *et al*., reported that local anesthesia into the unilateral inferior alveolar nerve, which decreases cognitive function, significantly reduced the induction of BBF by chewing^34^, suggesting oral somatosensory input from the PDL during chewing was important in the induction of BBF. Clinical studies have revealed the relationship between poor dental health status and the onset of dementia^35–37^. Occlusal stimuli from the PDL during chewing or mastication play an important role in the maintenance of favorable BBF and brain function. Together, impaired occlusal function in MP would negatively influence brain function.

Our results from EMG analyses clearly demonstrated diminished masseter and temporal muscle activity in MP as compared with NORM during clenching. Hasegawa *et al*., reported that contraction of masticatory muscles influences cerebral blood flow^38^, thus, attenuated masseter and temporal muscle activity in MP would also negatively influence brain function. Together, these reduced functions in masticatory muscles in MP would also negatively influence brain function.

Case report exhibited the improvement of BBF by surgical orthodontic treatment for MP, which signifying that the surgical orthodontic treatment for MP would improve brain function compromised by malocclusion.

In conclusion, we have found that MP results in attenuated BBF increase by chewing at the inferior frontal gyrus and, as such, MP would have a negative impact on cognitive function.

## Methods

### Study participants

This cross-sectional study was approved by the Ethics Committee of Tsurumi University School of Dental Medicine (Approved number: 1316) and conformed to the principles of the Declaration of Helsinki. Written informed consent was obtained for all subjects before study commencement. This is a human observational study and we have conformed to STROBE guidelines.

Subjects with normal occlusion (NORM) and patients with MP were included in the present study. The NORM group (n = 17, 4 males and 13 females, age: median: 19.0, first quartile: 19.0, third quartile: 20.0 years) met the following inclusion criteria: no missing teeth other than the third molar; appropriate overjet and overbite (2 to 4 mm each; appropriate occlusion at the anterior tooth region); skeletal and dental midline deviation of 1.0 mm or less from facial midline (no significant lateral deviation of the jaws); no functional symptoms such as temporomandibular joint disorder; no history of orthodontic treatment; Angle Class I molar relationship (favorable antero-posterior relationship between maxillary and mandibular first molars); and normal intermaxillary relationship (ANB angle: 3.1 ± 2.5, mandibular plane to FH angle: 26.6 ± 6.7). The MP group consisted of 25 patients (6 males and 19 females, age: median: 21.0, first quartile: 18.0, third quartile: 25.0 years) of skeletal Class III (the condition of mandibular prognathism) that required orthognathic treatment at the Tsurumi University hospital. They were selected by the following inclusion criteria: no congenital abnormalities; no missing teeth other than the third molar; anterior crossbite; skeletal and dental midline deviation of 1.0 mm or less from facial midline; no functional symptoms such as temporomandibular joint disorder; no history of orthodontic treatment; Angle Class III molar relationship (the condition of the mandibular first molar exhibits anterior position as compared to the maxillary first molar); and skeletal Class III intermaxillary relationship in ANB angle (−2.7 ± 2.5). There was no statistical difference in median age between the two groups.

Calculation of the required sample size was performed with the use of the statistical power analys software, G*Power^39^. We set the parameters as follows; Effect size = 0.7, significance level = 0.05, power = 0.7. In this condition, the required total sample size was 38, and our total sample number (42) was above the estimated number.

### Examination for occlusal contact using silicone materials

Occlusal contact was recorded using vinyl polysiloxane impression material (PerfectIM systems; J Morita, Tokyo, Japan), scanned with computer scanner, and the number of the occlusal contacts were counted using ImageJ software.

### EMG analysis for masseter muscles

Skeletal Class III patients underwent an EMG recording as described previously^16^. Briefly, the electrodes (inter-electrode distance: 15 mm; NEC medical systems, Tokyo, Japan) were attached to skin over the bilateral superficial part of the masseter muscle and the anterior part of temporal muscle using electrocardiogram paste (CardioCleam; Nihon-Kohden, Tokyo, Japan). The ground electrode was attached to the right earlobe. Surface EMG signals of masseter and anterior part of temoral muscles during the clenching task were obtained. The EMG value of the person with normal occlusion was used from the paper published from our institute^16^.

### fNIRS

**Figure 4:**
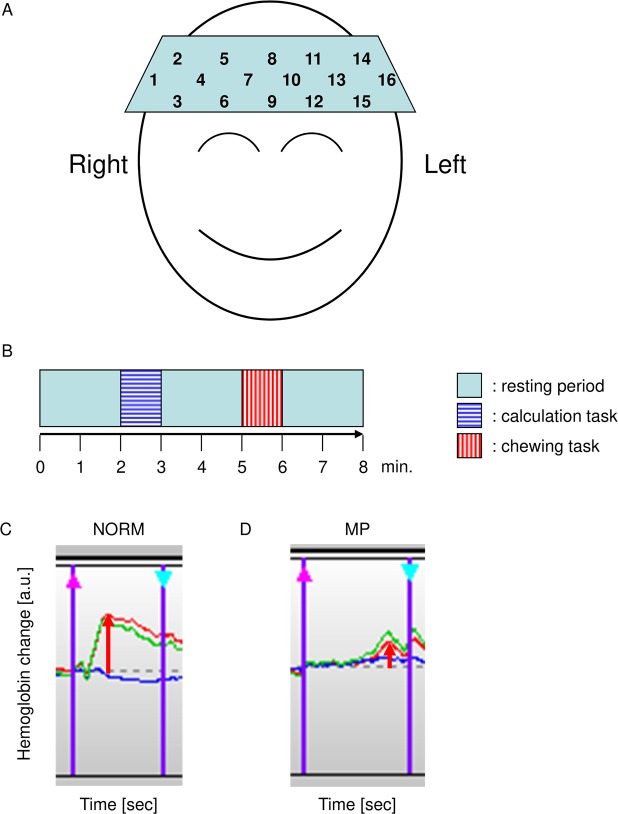
Examination of brain blood flow by functional near-infrared spectroscopy. (**A**) Probe position. (**A**) 16-channel probe matrix on Fpz (midpoint between Fp1 and Fp2) in accordance with the international 10/20 system used in electroencephalography. The probe in the bottom left corner was placed around F7, and the right probe was placed around F8. (**B**) Schematic illustrating the design of the fNIRS experiment. The calculation task (blue horizontal striped bar) and the chewing task (red vertical striped bar) were performed by all subjects. Adequate resting intervals of over 30 seconds were taken between each task. (**C**,**D**) fNIRS data showing the change in hemoglobin signals during the chewing task in channel-16, are shown on the same scale along the Y-axis. Representative data of NORM(C) and MP(D) are shown. Oxy-Hb (red), deoxy-Hb (blue), and total-Hb (green) were measured. X-axis indicates the time, and Y-axis indicates the change in hemoglobin signals. Magenta arrow on the left side indicates the start point of the task, and pale blue arrow on the right side indicates the end point of the task. In the present study, the maximum value of each task, as indicated by red arrow, were used in further analyses.

In the present study, fNIRS (OEG-16 apparatus: Spectratech, Tokyo, Japan) was used to detect BBF. This system is able to measure changes in oxygenated hemoglobin (oxy-Hb) concentration in the cortex of frontal lobe. Measurement of Hb changes was performed with 16 channels (Fig. 4A). The center of the probe matrix was placed on Fpz (midpoint between Fp1 and Fp2) in accordance with the international 10/20 system used in electroencephalography^40^. fNIRS can measure BBF non-invasively, and it is accurate enough^23,41,42^ to use clinically for diagnosis of depression and schizophrenia^43^. The intra-class correlation coefficients (ICC) by comparing the data of two time points with a week interval were over 0.7 in this study.

### Task setting for fNIRS

During fNIRS measurements, we used a simple block design consisting of control and experimental task conditions, with adequate resting intervals of over 30 seconds (Fig. 4B). The control task was a calculation task, in which the subjects were asked to verbally calculate the serial subtraction of 7 from 100. The experimental task was to chew CRT paraffin (Ivoclar vivadent, Tokyo, Japan) on their preferred side.

### fNIRS data analyses

Changes in BBF were inferred by oxy-Hb values at 16 channels, as oxy-Hb has been shown to be the most sensitive indicator of BBF changes in animal studies^44^. Baseline correction and moving averages were calculated to eliminate artifacts^45^. The maximum values of oxy-Hb in each channel during each task were used for the analysis (Fig. 4C,D). Groups were compared by calculating the ratio^46^ of oxy-Hb during the chewing task with the mean oxy-Hb value during the calculation task.

### Statistical analysis

Data were firstly tested for a normal distribution using a D’Agostino’s K-squared test. In a set of normally distributed samples, statistical significance was tested using Student’s *t*-test. Non-parametric tests were performed using a Mann-Whitney U analysis. Parametric data are expressed as mean ± standard error (SE), and non-parametric data are expressed as median and quartiles. A value of *P* < 0.05 was considered statistically significant.
